# Perceived eating norms and children's eating behaviour: An informational social influence account

**DOI:** 10.1016/j.appet.2017.02.015

**Published:** 2017-06-01

**Authors:** Maxine Sharps, Eric Robinson

**Affiliations:** University of Liverpool, Institute of Psychology, Health and Society, Eleanor Rathbone Building, University of Liverpool, Liverpool, L69 7ZA, United Kingdom

**Keywords:** Social norms, Social eating, Perceived eating norms, Food intake

## Abstract

There is initial evidence that beliefs about the eating behaviour of others (perceived eating norms) can influence children's vegetable consumption, but little research has examined the mechanisms explaining this effect. In two studies we aimed to replicate the effect that perceived eating norms have on children's vegetable consumption, and to explore mechanisms which may underlie the influence of perceived eating norms on children's vegetable consumption. Study 1 investigated whether children follow perceived eating norms due to a desire to maintain personal feelings of social acceptance. Study 2 investigated whether perceived eating norms influence eating behaviour because eating norms provide information which can remove uncertainty about how to behave. Across both studies children were exposed to vegetable consumption information of other children and their vegetable consumption was examined. In both studies children were influenced by perceived eating norms, eating more when led to believe others had eaten a large amount compared to when led to believe others had eaten no vegetables. In Study 1, children were influenced by a perceived eating norm regardless of whether they felt sure or unsure that other children accepted them. In Study 2, children were most influenced by a perceived eating norm if they were eating in a novel context in which it may have been uncertain how to behave, as opposed to an eating context that children had already encountered. Perceived eating norms may influence children's eating behaviour by removing uncertainty about how to behave, otherwise known as informational social influence.

## Highlights

• Mechanisms underlying the influence of perceived eating norms were examined.
• Perceived eating norms influenced children's vegetable consumption.
• Perceived eating norms may act as a form of informational social influence on consumption.

## 1 Introduction

A substantial body of literature suggests that eating behaviour can be socially influenced. People have been shown to adapt their eating behaviour to that of a present dining companion (Bevelander et al., 2012, Hermans et al., 2009, Robinson et al., 2013). Moreover, beliefs about the eating behaviour of others, otherwise known as perceived eating norms, have been consistently shown to influence eating behaviour in laboratory studies (Pliner and Mann, 2004, Robinson, 2015, Sharps and Robinson, 2015). For example, a number of studies showed that people eat more when exposed to information that suggests other people have eaten a large amount of food, compared to when exposed to information that suggests other people have eaten a small amount (Pliner and Mann, 2004, Robinson, 2015, Robinson et al., 2013, Robinson et al., 2014b).

The mechanisms that explain why perceived eating norms influence behaviour have received less attention. One explanation is that perceived eating norms may act as a form of normative social influence (Deutsch & Gerard, 1955), whereby people may copy the behaviour of others when they are concerned with feeling socially accepted or establishing a relationship with the source of the influence (Cialdini and Goldstein, 2004, Cialdini and Trost, 1998, Deutsch and Gerard, 1955). Humans have a desire to be liked by others and belong (Baumeister & Leary, 1995), and there is evidence that normative social influence may be a possible explanation for why people adjust their own food intake to the intake of a present peer (Hermans et al., 2009, Robinson et al., 2011). For example, Hermans, Engels, et al. (2009) found that participants only imitated the eating behaviour of a confederate when the confederate behaved in a ‘cold’ manner towards them, suggesting that participants may have imitated eating behaviour in order to persaude the confederate to accept them. In another study, Robinson et al. (2011) found that when participants were primed to feel socially accepted, they were less likely to match the intake of a confederate. This research linking normative explanations to social imitation of eating has predominantly focused on experimental paradigms which involve people eating together, however, there is also evidence that eating behaviour may be socially influenced due to a desire to ‘fit in’ even when peers are not present (Cruwys et al., 2012, Guendelman et al., 2011). For example, in one study (Cruwys et al., 2012) University students encountered a confederate, and were exposed to the popcorn intake of the confederate before being left alone to eat popcorn. Cruwys et al. (2012) found that the participants only adjusted their intake based on what they believed the confederate had eaten when they were led to believe that the confederate was from the same University as them (Cruwys et al., 2012). In addition, in two studies (Guendelman et al., 2011) Asian American participants were more likely to report prototypical American food as their favourite, and ordered and ate more American dishes after their American identity was challenged compared to when their identity was not challenged. Thus, these studies indicate that social factors may influence eating as a result of a desire to ‘fit in’. However, little other research has examined whether normative social influence may be a potential mechanism underlying the influence that perceived eating norms have on eating behaviour. Although research has shown that perceived eating norms influence eating behaviour (Pliner and Mann, 2004, Robinson, 2015, Robinson et al., 2013, Robinson et al., 2014b, Roth et al., 2001), at present we do not know whether people are influenced by perceived eating norms due to people wanting to ‘fit in’ and feel accepted, but it is a plausible explanation which warrants testing.

An alternative explanation to a normative account of social influence is that perceived eating norms may act as a form of informational social influence (Deutsch & Gerard, 1955). According to Cialdini and Trost (1998) people are often uncertain about how to behave in a situation, and other people's behaviour may act as a guide to determine the most appropriate course of action. Therefore, perceived eating norms which provide information about the eating behaviour of others may indicate the correct way to behave in a situation, e.g. ‘if a lot of people are doing this, it's probably a wise thing to do’ (Cialdini, 2007). Thus, conforming to the norm may be a way of reducing uncertainty in a situation, rather than other motives such as social acceptance or wanting to ‘fit in’(Cialdini and Goldstein, 2004, Deutsch and Gerard, 1955). As discussed, adults have been shown to be influenced by perceived eating norms (Pliner and Mann, 2004, Robinson, 2015, Robinson et al., 2013, Robinson et al., 2014b, Roth et al., 2001). Within these studies participants were typically exposed to perceived eating norms that suggested how others behaved in the same context (i.e. other participants in this study ate this amount of food) during a single experimental session. Since the research environment is likely to be novel and unfamiliar to the participants, it is feasible that perceived eating norms have a consistent effect on behaviour in these paradigms because they inform participants about the correct way to behave in the novel and unfamiliar eating context participants find themselves in. Therefore, it is not clear whether people are strongly influenced by perceived eating norms within these studies because the eating context may be unfamiliar and novel, or whether people would also be influenced by perceived eating norms if they have eaten in that context previously. If an informational social influence-based account of perceived eating norms is correct, then we would hypothesise that people would be most influenced by perceived eating norms when they find themselves in a novel context vs. a context they have previously eaten in. This is because people would be more uncertain about how to behave or ‘act’ in a novel context, as opposed to a context that a person has previously eaten in. Thus, understanding whether perceived eating norms influence behaviour to a greater extent in novel and unfamiliar contexts, as opposed to a familiar eating context is one approach by which to test an informational social influence account.

Although there is now reliable evidence that perceived eating norms influence eating behaviour in adults (Robinson et al., 2014b), less research has examined this in children (Sharps & Robinson, 2015). In one study, Sharps and Robinson (2015) exposed children to a perceived eating norm that outlined the vegetable intake of previous (fictitious) children in that study. Consistent with the adult literature, the children were influenced by the perceived eating norm, eating more when exposed to information suggesting that previous children had eaten a large amount, compared to when exposed to information suggesting that previous children had eaten no vegetables. As this is the only study to our knowledge which has directly investigated the influence of perceived eating norms on children's eating behaviour, further research is needed to replicate this effect. Furthermore, although research has started to examine evidence for mechanisms underlying social influences on eating behaviour in adults (Hermans et al., 2009, Robinson et al., 2011), less research has examined evidence for the mechanisms underlying the influence of perceived eating norms on children's eating behaviour.

The present research had two aims: Our first aim was to replicate the effect that perceived eating norms have on children's vegetable consumption (Sharps & Robinson, 2015). Our second aim was to examine evidence for possible mechanisms underlying the influence of perceived eating norms in children. In Study 1 we examined whether perceived eating norms may act as a form of normative social influence, whereby, children may be motivated to conform to a perceived eating norm in order to maintain personal feelings of social acceptance and ‘fit in’. In Study 2 we examined whether perceived eating norms may act as a form of informational social influence, by shaping eating behaviour when there is uncertainty about how to behave.

## 2 Study 1

### 2.1 Method

#### 2.1.1 Participants

100 children (53% females, 88% normal-weight) aged 6–11 years old (9.6 years, SD = 1.5) were recruited from two Primary schools in North-West England. Children were led to believe that the study was looking at how children play games. In recent work, we examined the effect of perceived eating norms on children's vegetable consumption and in this study we observed a statistically large effect (Sharps & Robinson, 2015). Therefore, sample sizes of 25 children per condition provided adequate statistical power to detect similar sized main effects of perceived eating norms in the present studies. Study 1 and 2 were approved by the University of Liverpool Research Ethics Committee. Fully-informed consent was provided and children with allergies or a history of allergies were unable to participate in both studies.

#### 2.1.2 Study overview

Children attended a single experimental session at a primary school. Children were either primed with feelings of peer acceptance, or with feelings of ambiguity about their peer acceptance. Next, children were exposed to information that indicated the vegetable consumption of previous (fictitious) children in the study (perceived eating norm). Dependent on condition, children either saw that previous children had eaten a large amount of vegetables, or no vegetables. All children were provided with a bowl of vegetables (carrots), and were left for 7 min to consume as much or as little as they liked. This design allowed us to examine whether children would be more likely to be influenced by a perceived eating norm if they were primed with feelings of ambiguity about peer acceptance, than when they were primed with feelings of peer acceptance.

#### 2.1.3 Experimental design

Children were randomised (using an online research randomiser; www.randomizer.org) into a 2 × 2 between-subjects design, with factors social influence condition (high vs. no intake) and peer acceptance condition (peer acceptance vs. ambiguity of peer acceptance).

#### 2.1.4 Social influence condition

Children were exposed to a fictitious participant information sheet that contained information about six previous participants (participant number, date of birth, gender). The fictitious participant information sheet contained four columns; participant number, date of birth, gender, and Carrots (amount eaten). The ‘Carrots (Amount eaten) column stated ‘all’ in the high intake condition, and ‘none’ in the no intake condition. Children were also presented with a bowl that appeared to be that of a previous participant. The bowl contained a single remaining carrot in the high intake condition, or was full in the no intake condition, to corroborate the fictitious participant information sheet.

#### 2.1.5 Priming peer acceptance or ambiguity of peer acceptance

We based our manipulation on previous work by Over and Carpenter (2009). First the researcher discussed what being ‘especially liked’ meant with the child i.e. ‘especially liked children are liked by other children, other children want to play with them, and they are always included in all of the games’. Next, every child was presented with a peer acceptance image that showed four cartoon children who were smiling and holding hands. The researcher pointed out that, in this image, one of the children was especially liked, and asked the child to explain what they thought this meant. Next, the researcher explained that not everyone can be especially liked and presented the child with the peer exclusion image, which showed the same four cartoon children. Three of the cartoon children were holding hands, and one was away from the group. The researcher asked the child to explain what they thought was happening in the image. Following this, the researcher explained that they have tried to work out who they think the children in the school are who are ‘especially liked’ by other children.

*Peer acceptance:* In the peer acceptance condition, the researcher explained that they believed that the child was especially liked (i.e. “From what I found out, I think that you are one of the types of children who are especially liked. Other children want to play with you and be your friend”). The researcher asked the child to describe what being especially liked meant.

*Ambiguity of peer acceptance:* In the ambiguity of peer acceptance condition the researcher explained that they would inform the child about whether they thought the child was especially liked after a short break.

Following exposure to the social influence condition manipulation (described above), all children were presented with a peer acceptance scale. In the peer acceptance condition, the researcher reiterated that they thought that the child was especially liked (“as I said, I think you are one of the especially liked children who everyone likes and wants to play with”) and placed a counter (‘You’) under the peer acceptance image. In the ambiguity of peer acceptance condition, the researcher reiterated that they would inform the child after the break whether the researcher thought they were especially liked. The researcher then placed the counter (‘You’) under ‘unsure’ on the scale.

### 2.2 Measures

#### 2.2.1 Fruit and vegetable consumption and liking

To assess usual fruit and vegetable consumption, the Day in the Life questionnaire was administered, which is a valid and reliable twenty-four hour recall measure for use in children (Edmunds & Ziebland, 2002). We included questions about children's liking of carrots (e.g. how much do you like the carrots you were given? And how much do you like carrots in general?), with 5 response options ranging from ‘not at all’ to ‘a lot’. These questions were assessed using smiley-face Likert-style scales and were based on questions used by Sharps and Robinson (2015).

#### 2.2.2 Body weight

Height was measured to the nearest 0.5 cm using a Stadiometer (Seca 213, Seca GmbH & Co.) and weight was measured to the nearest 0.1 kg using a digital scale (Seca 813 digital scale, Seca, GmbH & Co.). BMI was calculated as weight (kg)/height (m^2^). Using internationally recognised criteria for children (Cole & Lobstein, 2012) healthy-weight, overweight and obesity were defined based on age and sex-specific BMI cut-off points equivalent to adult BMI of 25–30kg/m^2^ respectively.

#### 2.2.3 Manipulation checks

To examine whether the social influence condition manipulation was successful, children were asked ‘how many carrots do you think other children ate in the study’, and were presented with three choices ‘none’, ‘some’, and ‘almost all’, alongside a photograph of either a full, half full, or empty bowl of carrots.

To examine whether the peer acceptance manipulation was successful, i.e. it caused children to believe that they were either accepted by their peers or were uncertain about whether they were accepted by their peers, children were asked ‘how especially liked do you think you are?’ and children were presented with a paper version of the peer acceptance scale, which was a 3-point scale which contained the peer acceptance image as one anchor, the peer exclusion image as the other anchor, and ‘unsure’ in the middle.

#### 2.2.4 Procedure

Children were tested individually during weekdays between 9am and 3.30 pm at a primary school. Children were informed that the researcher was interested in how children play games. First the child was primed with feelings of peer acceptance or ambiguity of peer acceptance. Following this, the child was presented with the fictitious participant information sheet, and completed the date of birth and gender columns with the researcher. The researcher pointed out the ‘Carrots (amount eaten)’ column and explained that this did not need to be completed, and had only been completed previously for carrot buying purposes. The researcher then pointed out the intake of previous children. In all conditions the researcher ‘noticed’ the bowl on the table and described the intake of previous children to the child. Next, the child was presented with a bowl of vegetables (carrots). At this point the child was presented with the peer acceptance scale as described in the priming procedure. Next, every child was then presented with a paper version of the peer acceptance scale and asked to indicate how especially liked they believed they were. The researcher then explained that they would leave the child alone while the researcher sorted out the game and that they could eat as much or as little of the snack as they wished. The child was left alone for 7 min to eat as many or as few vegetables as they wished. After the 7 min, the researcher returned. In children primed with ambiguity of peer acceptance the researcher then explained to the child that they believed that the child was especially liked. To corroborate the cover story all children were then presented with the game and the researcher explained that the game involved trying to find pairs of animal images. Both bowls were removed from the table and the child was left to play the game for three minutes. Finally, the researcher asked the child what they thought the aims of the study were, and completed the remaining questionnaire measures with the child. Height and weight were subsequently measured.

#### 2.2.5 Analysis strategy

The main planned analysis was a 2 × 2 ANOVA, with factors social influence condition (high vs. no intake) and peer acceptance condition (peer acceptance vs. ambiguity of peer acceptance). The dependent variable was children's vegetable consumption (in grams). We planned to follow up significant effects of the manipulation checks and main analyses with Bonferroni-corrected pairwise comparisons.

### 2.3 Results

No differences (*p*s > 0.05) were found between the conditions for age, gender or BMI. See Table 1.

#### 2.3.1 Manipulation checks

No children guessed, or came close to guessing the aims of the study. To check whether children believed the manipulations, 2 × 2 ANOVAs were conducted on children's beliefs about the amount of vegetables (carrots) eaten by other children, and on children's beliefs about how socially accepted they believed they were.

##### 2.3.1.1 Social influence condition manipulation

There was a significant main effect of social influence condition on children's beliefs about the amount of vegetables eaten by other children [F (1, 96) = 130.22, *p* < 0.001, ƞp^2^ = 0.58]. There was no significant main effect of peer acceptance condition on children's beliefs about the amount of vegetables eaten by other children [F (1, 96) = 2.66, *p* = 0.106, ƞp^2^ = 0.03]. However, a significant social influence condition x peer acceptance condition interaction was observed [F (1, 96) = 5.98, *p* = 0.016, ƞp^2^ = 0.06]. We therefore examined the effect of social influence condition on children's beliefs about the amount of vegetables eaten by other children in the peer acceptance vs. ambiguity of peer acceptance conditions separately.

In the peer acceptance condition, independent samples t-tests revealed that children exposed to the high intake norm believed that other children had eaten more vegetables (n = 25, *M* = 2.48, SD = 0.51) than did children who were exposed to the no intake norm (n = 25, *M* = 1.12, SD = 0.33), t (48) = 11.18, *p* < 0.001, *d* = 3.17. In the ambiguity of peer acceptance condition, independent samples t-tests also revealed that children exposed to the high intake norm believed that other children had eaten more vegetables (n = 25, *M* = 2.40, SD = 0.58) than did children exposed to the no intake norm (n = 25, *M* = 1.52, SD = 0.51), t (48) = 5.71, *p* < 0.001, *d* = 1.61. Thus, in both peer acceptance conditions children exposed to the high intake norm believed that previous children in the study had eaten more vegetables than children exposed to the no intake norm. However, the social influence condition manipulation had a stronger effect in children primed with peer acceptance vs. ambiguity of peer acceptance.

##### 2.3.1.2 Peer acceptance manipulation

There was a significant main effect of peer acceptance condition on children's beliefs about how especially liked they believed they were [F (1, 96) = 10.87, *p* = 0.001, ƞp^2^ = 0.10]. Children in the peer acceptance condition reported feeling more especially liked (n = 50, *M* = 2.72, SD = 0.50), than children in the ambiguity of peer acceptance condition (n = 50, *M* = 2.38, SD = 0.53). 74% of children in the peer acceptance condition believed that they were especially liked in comparison to 40% of children in the ambiguity of peer acceptance condition. There was no significant main effect of social influence condition [F (1, 96) = 0.34, *p* = 0.562, ƞp^2^ = 0.004], and no significant peer acceptance condition x social influence condition interaction was observed on children's beliefs about how especially liked they believed they were [F (1, 96) = 0.94, *p* = 0.335, ƞp^2^ = 0.01].

#### 2.3.2 Vegetable consumption

**Fig. 1:**
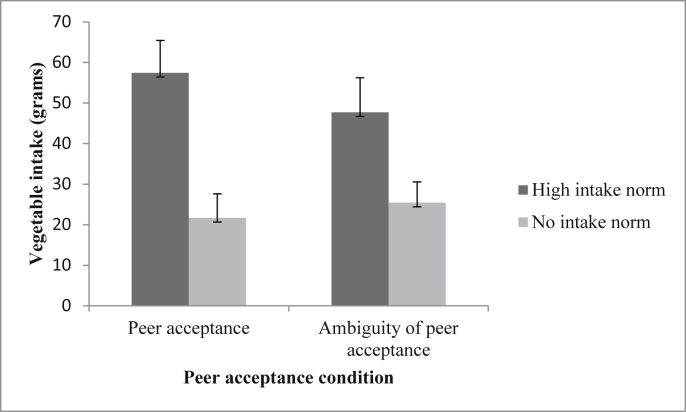
Mean (±SEM) vegetable consumption (in grams) as a function of peer acceptance condition and social influence condition.

Using a 2 (social influence condition) x 2 (peer acceptance condition) between-subjects ANOVA, there was a significant main effect of social influence condition on children's vegetable consumption (in grams) [F (1, 96) = 16.93, *p* < 0.001, ƞp^2^ = 0.15]. Children in the high intake conditions ate significantly more vegetables than children in the no intake conditions. There was no significant main effect of peer acceptance condition on children's vegetable consumption [F (1, 96) = 0.18, *p* = 0.671, ƞp^2^ = 0.002], and no significant social influence condition x peer acceptance condition interaction was observed on children's vegetable consumption [F (1, 96) = 0.92, *p* = 0.340, ƞp^2^ = 0.009]. See Fig. 1 for mean vegetable consumption values.

#### 2.3.3 Other variables

Controlling for *z*BMI, child age, liking of carrots, and usual fruit and vegetable intake as covariates in separate 2 (social influence condition) x 2 (peer acceptance condition) ANCOVAs, and including gender in the analyses did not alter the results of the analyses examining children's vegetable consumption.

### 2.4 Discussion

Consistent with a previous study (Sharps & Robinson, 2015), the results of Study 1 showed that children were influenced by perceived eating norms regarding other children's vegetable consumption, eating more vegetables when they were led to believe that previous children had eaten a large amount of vegetables compared to when they were led to believe that previous children had eaten no vegetables. However, regardless of whether children were primed with feelings of peer acceptance or feelings of ambiguity of peer acceptance, children were similarly influenced by the perceived eating norm. The results do not support our hypothesis that priming children with feelings of peer acceptance may reduce the influence of a perceived eating norm relative to priming children with feelings of ambiguity of peer acceptance. In Study 2, we aimed to test whether perceived eating norms may act as a form of informational social influence, providing a guide for how to behave in a novel and unfamiliar eating context (Cialdini and Trost, 1998, Deutsch and Gerard, 1955). We hypothesised that children would be strongly influenced by a perceived eating norm in a novel and unfamiliar context, but be less influenced when eating in a familiar eating context they had encountered before.

## 3 Study 2

### 3.1 Method

#### 3.1.1 Participants

Due to the repeated measures design in Study 2, we were conscious of potential dropout, and therefore opted to recruit a minimum of 30 children per experimental condition. 131 children were recruited from three Primary schools in the North-West of England. One child was excluded due to not being available for both study sessions and three children were excluded as they were unable to understand the study instructions. The final sample consisted of 127 children (54.3% females) aged 6–11 years old (M = 8.32, SD = 1.30).

#### 3.1.2 Study overview

Children participated in two sessions, one day apart. Children were exposed to information about the about the vegetable consumption of previous children in the study in one of the sessions (perceived eating norm), and received no information about the vegetable consumption of previous children in the study in the other session. Dependent on condition children either saw the perceived eating norm during their first session (unfamiliar eating context) or in their second session (familiar eating context). As in Study 1, dependent on condition, the perceived eating norm either indicated that previous children in the study had eaten a large amount of vegetables or no vegetables. Children were given a bowl of vegetables (carrots) to eat in both sessions and their vegetable consumption was examined in both sessions. In line with an informational social influence hypothesis, this design allowed us to test whether children would be more strongly influenced by a perceived eating norm in a novel and unfamiliar context, but be less influenced when eating in a familiar eating context that they had encountered before. Because of the design of the study we were also able to examine whether being exposed to perceived eating norm information during session 1 continued to affect vegetable consumption a day later (session 2) in the absence of that perceived eating norm information.

#### 3.1.3 Experimental design

Participants were randomised into a 2 × 2 x 2 mixed design, with between subjects' factors; social influence condition (high vs. no intake) and familiarity of the eating context condition (familiar vs. unfamiliar), and a within subject's factor of eating session (session 1 and session 2). Study 2 adopted the same remote-confederate design as Study 1, whereby children were exposed to the same fictitious participant information sheet and a bowl which suggested that other children either ate a large amount of vegetables or no vegetables during one of the two sessions they participated in. In the session in which children were not exposed to social influence condition information, the column ‘Carrots (Amount eaten)’ remained blank, and the bowl contained an item unrelated to food (pens).

#### 3.1.4 Explanation of familiarity of the eating context condition

In order to manipulate familiarity of the eating context, we manipulated the session in which children were exposed to the social influence condition information. In the ‘unfamiliar eating context’ condition, children were exposed to the social influence condition information in session 1, and received no intake information in session 2 (see above). In the ‘familiar eating context’ condition children were exposed to the social influence condition information in session 2, and saw no intake information in session 1.

#### 3.1.5 Measures

The measures were the same as in Study 1. However, we included a hunger measure in Study 2. Hunger was measured using a child hunger scale developed by Bennett and Blissett (2014). Response options ranged from ‘very hungry’ to ‘not hungry at all/very full’ (Bennett & Blissett, 2014).

#### 3.1.6 Manipulation check

The same social influence condition manipulation check was used as Study 1.

#### 3.1.7 Procedure

##### 3.1.7.1 Session 1

Children were tested individually between 9am and 3.30 pm at a Primary school. The sessions took place one day apart, at approximately the same time. Children were informed that the study involved two sessions and that the researcher was interested in whether playing a game in session 1 affected their performance in session 2. First, the researcher presented the child with the hunger measure, and the child was asked to rate how hungry they were. Next, the researcher presented the child with the fictitious participant information sheet. The researcher completed the date of birth and gender columns with the child. In the ‘unfamiliar eating context’ condition, the ‘Carrots (Amount eaten)’ column contained social influence condition information (i.e. it either stated ‘all’ or ‘none’ depending on which social influence condition the children were in). In the ‘familiar eating context’ condition this column was blank. In both conditions the researcher explained that the ‘Carrots (Amount eaten)’ column did not need to be completed and had only been completed previously for carrot buying purposes. In addition, in the ‘unfamiliar eating context’ condition the researcher pointed out the intake of the previous children. Next, in both conditions the researcher ‘noticed’ the bowl on the table and explained that it had been left there by accident. In the ‘unfamiliar eating context’ condition the bowl contained vegetables (i.e. was either full of carrots or contained a single remaining carrot to corroborate with the fictitious participant information sheet). In the ‘familiar eating context’ condition, the bowl contained an item unrelated to food (pens). In the ‘unfamiliar eating context’ condition the researcher described the intake of the previous children to the child. Next, in all conditions, the researcher explained to the child that they could have a snack while the researcher prepared the game. The researcher presented the child with the second bowl of carrots and explained to the child that they could eat as many as they wished. The fictitious participant information sheet and the first bowl remained on the table in all conditions. The child was left alone for 7 min to eat as many or as few carrots as they wished. After the 7 min, the researcher returned. The researcher removed the bowls and the fictitious participant information sheet from the table and presented the child with a game (the game involved matching two animal images to make a pair). The researcher explained how to play the game and the child was left to play the game for 3 min. On return, the researcher congratulated the child on their performance in the game to corroborate the cover story. Children in the ‘unfamiliar eating context’ condition completed the manipulation check to examine whether the social influence condition norm manipulation influenced children's beliefs about the amount of vegetables eaten by previous children.

##### 3.1.7.2 Session 2

Session 2 was identical to session 1. The only difference was that children in the ‘familiar eating context’ condition were now exposed to the social influence condition information (fictitious information sheet and bowl of carrots communicating the perceived eating norm), while children in the ‘unfamiliar eating context’ condition did not receive any social influence condition information and instead were exposed to the blank fictitious information sheet and bowl of pens. At the end of session 2, children in the ‘familiar eating context’ condition completed the manipulation check. All children were asked the aims of the study, and completed the remaining questionnaire measures with the researcher at the end of session 2. Weight and height were measured at the end of session 2.

#### 3.1.8 Analysis strategy

The main planned analysis was a 2 × 2 x 2 mixed ANOVA, with between subjects factors familiarity of the eating context and social influence condition, and the within subjects factor of eating session. The dependent variable was children's vegetable consumption (in grams). As in Study 1, we planned to follow up significant effects of the manipulation checks and main analyses with Bonferroni-corrected pairwise comparisons. We hypothesised a significant eating session x social influence condition x familiarity of eating context interaction. We expected that exposure to the perceived eating norm in the novel eating context (i.e. session 1) would influence children's vegetable consumption, whereas, children's vegetable consumption may be less influenced following exposure to the norm in the familiar eating context (i.e. session 2). Including eating session as a factor was important due to the possibility that the social influence information in the unfamiliar eating context condition may spill over from session 1 to session 2, and to account for any other unpredicted effects of eating session.

### 3.2 Results

No differences (*p*s > 0.05) were found between the conditions for age, gender or *z*BMI. See Table 2.

#### 3.2.1 Manipulation check

No children guessed, or came close to guessing the aims of the study. To check whether children believed the norm manipulation, a 2 × 2 ANOVA was conducted on children's beliefs about the amount of vegetables (carrots) eaten by other children. There was a significant main effect of social influence condition on children's beliefs about the amount of vegetables eaten by other children [F (1, 123) = 132.23, *p* < 0.001, ƞp^2^ = 0.52]. There was no significant main effect of familiarity of eating context on children's beliefs about the amount of vegetables eaten by other children [F (1, 123) = 1.52, *p* = 0.221, ƞp^2^ = 0.01]. However, a significant social influence condition x familiarity of the eating context condition interaction was observed [F (1, 123) = 5.02, *p* = 0.027, ƞp^2^ = 0.04]. We therefore examined the effect of social influence condition on children's beliefs about the amount of vegetables eaten by other children in the familiar and unfamiliar eating contexts separately.

In the unfamiliar eating context, independent samples t-tests revealed that children who were exposed to the high intake norm believed that other children had eaten more vegetables (n = 32, *M* = 2.81, SD = 0.40) than did children who were exposed to the no intake norm (n = 33, *M* = 1.58, SD = 0.56), t (63) = 10.24, *p* < 0.001, *d* = 2.53. In the familiar eating context, independent samples t-tests also revealed that children exposed to the high intake norm believed that other children had eaten more vegetables (n = 32, *M* = 2.50, SD = 0.51) than did children exposed to the no intake norm (n = 30, *M* = 1.67, SD = 0.55), t (60) = 6.22, *p* < 0.001, *d* = 1.56. Thus, in both the familiar and unfamiliar eating contexts, children exposed to the high intake norm believed that other children had eaten more vegetables in comparison to children who were exposed to the no intake norm. However, the social influence condition manipulation had a stronger effect in children in the unfamiliar vs. familiar eating context.

#### 3.2.2 Vegetable consumption

Using a 2 (social influence condition) x 2 (familiarity of eating context) x 2 (eating session) mixed ANOVA, there was a significant main effect of social influence condition [F (1, 123) = 9.87, *p* = 0.002, ƞp^2^ = 0.07], no significant main effect of familiarity of eating context [F (1, 123) = 0.85, *p* = 0.359, ƞp^2^ = 0.007] and no significant main effect of eating session [F (1, 123) = 1.03, *p* = 0.313, ƞp^2^ = 0.01] on children's vegetable consumption (in grams). There were no significant interactions between social influence condition and familiarity of eating context [F (1, 123) = 2.81, *p* = 0.096, ƞp^2^ = 0.02], eating session and social influence condition [F (1, 123) = 0.29, *p* = 0.589, ƞp^2^ = 0.002], or eating session and familiarity of eating context on children's vegetable consumption [F (1, 123) = 0.04, *p* = 0.845, ƞp^2^ < 0.001]. However, as hypothesised, a significant eating session x social influence condition x familiarity of eating context interaction was observed [F (1, 123) = 7.18, *p* = 0.008, ƞp^2^ = 0.06]. We therefore examined the effects of social influence condition and eating session on children's vegetable consumption in the unfamiliar and familiar eating contexts separately.

##### 3.2.2.1 Unfamiliar eating context

**Fig. 2:**
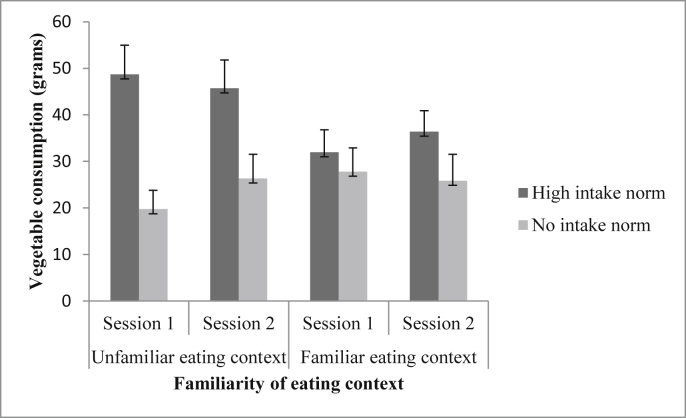
Mean (±SEM) vegetable consumption (in grams) as a function of familiarity of the eating context and social influence condition.

In the unfamiliar eating context, there was a significant main effect of social influence condition on children's vegetable consumption [F (1, 63) = 10.70, *p* = 0.002, ƞp^2^ = 0.15]. There was no significant main effect of eating session on children's vegetable consumption [F (1, 63) = 0.71, *p* = 0.402, ƞp^2^ = 0.01]. There was a significant eating session x social influence condition interaction [F (1, 63) = 5.05, *p* = 0.028, ƞp^2^ = 0.07]. Independent samples t-tests revealed that, in session 1, children who were exposed to the high intake norm ate significantly more vegetables than children who were exposed to the no intake norm, t (63) = 3.92, *p* < 0.001, *d* = 0.97. Furthermore, this effect persisted into session 2, whereby children who had been exposed to a high intake norm in session 1, ate significantly more vegetables in session 2 than children who had been exposed to a no intake norm in session 1, t (63) = 2.43, *p* = 0.036, *d* = 0.60. To explore this interaction further, paired samples t-tests were conducted to compare children's vegetable consumption in the high intake norm condition in session 1 vs. session 2, and to compare children's vegetable consumption in the no intake norm condition in session 1 vs. session 2. There were no significant between session differences in either the high intake, t (31) = 1.31, *p* = 0.400, *d* = 0.08, or the no intake norm condition, t (32) = 1.85, *p* = 0.148, *d* = - 0.24. See Fig. 2 for mean intake values. Thus, when children were exposed to a high vs. no intake norm in a context in which they had not previously eaten, their consumption of vegetables was affected by the norm information. This effect on behaviour persisted the next day when no social influence condition information was present, albeit to a lesser extent.

##### 3.2.2.2 Familiar eating context

In the familiar eating context there was no significant main effect of social influence condition [F (1, 60) = 1.19, *p* = 0.280, ƞp^2^ = 0.02] or eating session [F (1, 60) = 0.35, *p* = 0.558, ƞp^2^ = 0.01] on children's vegetable consumption. There was also no significant interaction between eating session and social influence condition on children's vegetable consumption [F (1, 60) = 2.36, *p* = 0.130, ƞp^2^ = 0.04]. Thus, when children were exposed to a high vs. no intake norm in a context they had previously eaten, their consumption of vegetables was not significantly affected.

#### 3.3.3 Other variables

Controlling for *z*BMI, hunger, child age, liking of carrots, and usual fruit and vegetable intake as covariates in separate 2 (social influence condition) x 2 (familiarity of eating context) x 2 (eating session) mixed ANCOVAs, and including gender in the analyses did not alter the results reported above.

## 4 General discussion

The present studies had two aims: First, we aimed to replicate the effect of perceived eating norms on children's vegetable consumption (Sharps & Robinson, 2015). Second, we aimed to examine the mechanisms that underlie why children are influenced by perceived eating norms. In both studies we found that children were influenced by perceived eating norms regarding other children's vegetable consumption, eating more vegetables when they were led to believe that previous children had eaten a large amount of vegetables, compared to when they were led to believe that previous children had eaten no vegetables. Study 1 showed that children were influenced by perceived eating norms regardless of whether they were primed with feelings of peer acceptance or ambiguity of peer acceptance. Study 2 showed that children were most strongly influenced by perceived eating norms when they were exposed to a norm in an unfamiliar eating context. Moreover, this effect persisted into a second session when eating norm information was not present. However, when children were exposed to the norm when they were in an eating context that they had previously eaten in, children's vegetable consumption was not significantly influenced. The results of Study 2 are consistent with the growing body of research which suggests that perceived eating norms may act as a form of informational social influence on eating behaviour when people are uncertain of how to behave (Herman and Polivy, 2005, Robinson et al., 2014b).

In Study 2 we found that an eating norm presented in a first session continued to influence children's eating behaviour in a session twenty-four hours later when the norm information was no longer present. This finding is consistent with a previous study investigating peer imitation of food intake in children (Bevelander et al., 2012). Herman and Polivy (2005) distinguish between situational and personal norms and suggest that situational norms are derived from the eating environment itself, such as the eating behaviour of others, whereas personal norms are based on an individual's prior experience. Consistent with Bevelander et al. (2012), we suggest that the perceived eating norms may have provided the situational norm in session 1, however, children may have then internalised this to be a personal norm and therefore behaved similarly in the second session. To our knowledge, little research has investigated the persistence of perceived eating norms over time. Further research is needed to examine whether perceived eating norms learnt in one context may ‘spill over’ and influence eating behaviour in different contexts, or whether the long-term influence of perceived eating norms observed in the present study is specific to the context in which the norm was ‘learnt’. Understanding this distinction may have important implications for interventions. If it is the case that a perceived eating norm ‘learnt’ in one context continues to influence eating behaviour only in that same context, then future intervention work would need to consider this.

In Study 1 we found little evidence that the influence a perceived eating norm (a norm about what others do) had on vegetable consumption was affected by ambiguity concerning social approval. A possible explanation for this may be the remote-confederate study design used in the present study, whereby the children were alone and no peers were present. Thus, it may be the case that the children did not feel a desire to ‘fit in’ without peers present. Another possible explanation is that since the norm information in the present studies described the behaviour of others (Cialdini, Reno, & Kallgren, 1990), they may not have provided information about what others approve of. Therefore, the children may not have been able to fulfil their social acceptance goals through adhering to this norm. One type of norm that may exert its influence through normative social influence is an injunctive norm. Injunctive norms provide information about what other people approve of (Cialdini et al., 1990). The influence injunctive norms have on eating behaviour is unclear. There is some evidence that injunctive norms are related to intentions to consume a healthy diet (Yun & Silk, 2011). Furthermore, there is evidence that injunctive norms may influence perceptions of the healthiness and tastiness of food carrying health halos (Vasiljevic, Pechey, & Marteau, 2015). For example, Vasiljevic et al. (2015) showed that a frowning emoticon label reduced participants' perceptions of the healthiness and tastiness of cereal bars. However, there is also evidence that injunctive norms reduce healthy eating intentions (Stok, De Ridder, De Vet, & De Wit, 2014), and in one study (Staunton, Louis, Smith, Terry, & Mcdonald, 2014) while an injunctive norm on its own did not influence intentions, when a negative descriptive norm was made salient, an injunctive norm reduced healthy eating intentions (Staunton et al., 2014). Furthermore, some studies have found little evidence that injunctive norms influence behaviour (Lally et al., 2011, Robinson et al., 2014a). It may be that when perceived injunctive norms do affect behaviour, they exert their influence through social approval concerns and further research is needed to examine this.

One factor which has been shown to affect whether eating behaviour is socially influenced is feelings of identification with the norm reference group (Berger and Rand, 2008, Cruwys et al., 2012). According to Festinger's (1954) social comparison theory and social identity theory (Hogg & Terry, 2016), people often evaluate themselves by comparing themselves to others, and are therefore more likely to follow the behaviour of similar others they identify with (Berger and Rand, 2008, Cruwys et al., 2012, Terry et al., 1999). For example, Berger and Rand (2008) showed that when participants were exposed to a perceived eating norm suggesting that an outgroup consumed junk food, participants were more likely to make healthy food choices. In another study, Cruwys et al. (2012) showed that adult participants were only motivated to adjust their intake to that of a previous participant when they were led to believe that the norm came from an ingroup rather than an outgroup member. In the present studies, we informed children that the perceived eating norm information referred to previous children in the study. While it was not explicitly stated that these were other children in the school, the nature of the study design indicated to the children that other children in their school had taken part. We did not measure how strongly participants in our studies identified with the other children in the school. Future studies could manipulate identification with the norm reference group in order to determine whether this affects the extent to which children are influenced by perceived eating norms.

An important consideration in the present studies is social context. In the present studies children were exposed to information about other children's eating behaviour in a very specific social context, i.e. other children ate like this in this study, and these context specific perceived eating norms had a statistically large effect on children's vegetable consumption. However, in two previous studies, Sharps and Robinson (2016) examined the effect of perceived eating norm messages about other children's eating habits, which were not specific to a particular social context, on children's fruit and vegetable consumption (Sharps & Robinson, 2016). The perceived eating norm messages were shown to only have a modest effect on children's eating behaviour (Sharps & Robinson, 2016). Research suggests that the influence that normative information has on behaviour decreases as norm based information becomes less specific to a given context (Goldstein, Cialdini, & Griskevicius, 2008). Hermans, Salvy, Larsen, and Engels (2012) showed that when participants were exposed to a video confederate who was in a different social context to the participant (i.e. in a university office (Study 1) or a living room (Study 2), participants did not adjust their intake to that of the video model. The authors suggested that this may be due to the participants finding themselves in a different social context to the video confederate. This point may be of importance, as the present studies only examined the influence of perceived eating norms in a very specific context and do not tell us about whether children's generalised beliefs about the eating behaviour of their peers influence their everyday eating behaviour.

In the present studies children believed the perceived eating norm manipulation, i.e. children exposed to the norm which suggested that previous children had eaten a large amount of vegetables, believed that other children had eaten more vegetables, than did children who were exposed to the norm which suggested that previous children had eaten no vegetables. However, in Study 1, children who were primed with peer acceptance more strongly believed the norm than children who were primed with ambiguity of peer acceptance. In Study 2, children who were presented with the norm in the unfamiliar eating context more strongly believed the norm than children presented with the norm in the familiar eating context. It is plausible that this pattern of results may be explained by the amount of attention children paid to the perceived eating norm information. In Study 1, children who were told they were socially accepted may have felt a stronger sense of identity with their fellow classmates and therefore attended more closely to the norm (Hogg and Reid, 2006, Turner et al., 1989). In Study 2, children who found themselves in an unfamiliar eating context may have been more likely to attend to the norm information because of uncertainty of how to behave. The latter interpretation is in fitting with the proposition that perceived eating norms may be particularly important in novel eating contexts. However, the between group differences we observed on our perceived eating norm manipulation check measures were unexpected in both studies. Understanding why these differences occurred will now be important.

The two studies presented here are the first to investigate mechanisms that may underlie the influence of perceived eating norms on children's vegetable consumption. However, the studies are not without limitations. The studies investigated whether perceived eating norms influenced children's carrot intake, therefore, it is not clear whether perceived eating norms will influence the intake of other, less liked vegetables. In Study 1 although we measured whether our manipulation to prime feelings of ambiguity of peer acceptance affected children's feelings of social acceptance we did not measure whether children were motivated to gain social approval. It may be the case that our manipulation was not strong enough to shift children's social approval motivation. Furthermore, the scale used to prime the children with feelings of peer acceptance or ambiguity was the same as the scale used to measure whether children believed the manipulation. While this may provide children with the opportunity to simply reproduce what they were told, in Study 1 our results indicate that this was not the case. However, using different measures to prime children and to measure the manipulation would be useful in future studies. In Study 2, although we manipulated whether an eating context was unfamiliar or familiar, we did not directly measure how uncertain children felt about how to behave in either eating context. Producing a measure which accurately taps into uncertainty may be particularly difficult in this age range, therefore we opted not to measure it in this instance. However, directly measuring uncertainty about how to behave and examining the effect this has on the influence of perceived eating norms would produce a more accurate test of an informational social influence hypothesis. Finally, here we examined evidence for the mechanisms in two separate studies, it would however, be useful to pit the two mechanisms against each other in a single study.

In conclusion, across two studies we provide further evidence that children are influenced by perceived eating norms regarding other children's vegetable consumption. Moreover, we suggest that perceived eating norms may exert their influence on eating behaviour through informational social influence.

## Funding

ER was partly supported by the Wellcome Trust (097826/Z/11/A).

## Conflicts of interest

The authors report no conflicts of interest.

## Figures and Tables

**Table 1:** Mean values (SDs) and statistical test results for BMI, age and gender for Study 1.

Variables	Peer acceptance (n = 50)		Ambiguity of peer acceptance (n = 50)		Test statistic and *p*-value
	High intake (n = 25)	No intake (n = 25)	High intake (n = 25)	No intake (n = 25)	
BMI (*z*-score)	0.08 (1.44)	0.15 (0.96)	0.14 (1.20)	0.34 (1.08)	Social influence condition: F (1, 96) = 0.33, *p* = 0.565, ƞp^2^ = 0.003.
					Peer acceptance condition: F (1, 96) = 0.26, *p* = 0.609, ƞp^2^ = 0.003.
					Social influence condition x peer acceptance condition interaction: F (1, 96) = 0.08, *p* = 0.785, ƞp^2^ = 0.001.
Age (years)	9.58 (1.48)	9.54 (1.48)	9.78 (1.59)	9.57 (1.58)	Social influence condition: F (1, 96) = 0.17, *p* = 0.681, ƞp^2^ = 0.002
					Peer acceptance condition: F (1, 96) = 0.13, *p* = 0.724, ƞp^2^ = 0.001.
					Social influence condition x peer acceptance condition interaction: F (1, 96) = 0.08, *p* = 0.784, ƞp^2^ = 0.001.
Gender					
Boys (n)	14	12	10	11	X^2^ (3, n = 100) = 1.41, *p* = 0.704, *r* = 0.12.
Girls (n)	11	13	15	14	

**Table 2:** Mean values (SDs) and statistical test results for BMI, age and gender for Study 2.

Variables	Unfamiliar eating context (n = 65)		Familiar eating context (n = 62)		Test statistic and *p*-value
	High intake (n = 32)	No intake (n = 33)	High intake (n = 32)	No intake (n = 30)	
BMI (*z*-score)	0.21 (1.23)	0.15 (1.05)	0.27 (1.04)	0.17 (1.20)	Social influence condition: F (1, 123) = 0.15, *p* = 0.704, ƞp^2^ = 0.001
					Familiarity of eating context: F (1, 123) = 0.03, *p* = 0.853, ƞp^2^ < 0.001.
					Social influence condition x familiarity of eating context interaction: F (1, 123) = 0.01, *p* = 0.933, ƞp^2^ < 0.001.
Age (years)	8.36 (1.25)	8.20 (1.28)	8.40 (1.41)	8.30 (1.31)	Social influence condition: F (1, 123) = 0.31, *p* = 0.581, ƞp^2^ = 0.002.
					Familiarity of eating context: F (1, 123) = 0.09, *p* = 0.764, ƞp^2^ = 0.001.
					Social influence condition x familiarity of eating context interaction: F (1, 123) = 0.01, *p* = 0.920, ƞp^2^ < 0.001.
Gender					
Boys (n)	15	15	11	16	X^2^ (3, n = 127) = 2.35, *p* = 0.503, *r* = 0.14.
Girls (n)	17	18	21	14	
